# Polymer Electrolytes Based on Na-Nafion Plasticized by Binary Mixture of Ethylene Carbonate and Sulfolane

**DOI:** 10.3390/membranes12090840

**Published:** 2022-08-29

**Authors:** Anna A. Krupina, Ruslan R. Kayumov, Grigory V. Nechaev, Alexander N. Lapshin, Lyubov V. Shmygleva

**Affiliations:** 1 Moscow Institute of Physics and Technology, 9 Institutskiy per., Dolgoprudny 141701, Moscow Region, Russia; 2 Institute of Problems of Chemical Physics of Russian Academy of Sciences, 1 Academician Semenov Av., Chernogolovka 142432, Moscow Region, Russia

**Keywords:** Nafion, sodium, polymer electrolyte, sulfolane, ethylene carbonate, aprotic solvent, ionic conductivity, thermal analysis, IR spectroscopy

## Abstract

The development of post-lithium current sources, such as sodium-ion batteries with improved energy characteristics and an increased level of safety, is one of the key issues of modern energy. It requires the search and study of materials (including electrolytes) for these devices. Polyelectrolytes with unipolar cationic conductivity based on Nafion^®^ membranes are promising. In this work, the effect of swelling conditions of the Nafion^®^ 115 membrane in Na^+^-form with mixtures of aprotic solvents such as ethylene carbonate and sulfolane on its physicochemical and electrotransport properties was studied. Nafion-Na^+^ membranes were swollen in a mixture of solvents at temperatures of 40, 60, and 80 °C. The results were obtained using methods of impedance spectroscopy, simultaneous thermal analysis, and IR spectroscopy. The best conductivity was observed for a membrane swelling at 80 °C in a mixture with a mass fraction of ethylene carbonate of 0.5, which reaches 10^−4^ S cm^−1^ at 30 °C and retains rather high values down to −60 °C (10^−6^ S cm^−1^). Thus, it is possible to expand the operating temperature range of a sodium battery by varying the composition of the polymer electrolyte and the conditions for its preparation.

## 1. Introduction

The development and research of new fire- and explosion-proof electrochemical systems with high energy density and operability over a wide temperature range is one of the key issues of modern power engineering. Along with the expansion of the market for electrical appliances (such as smartphones, laptops, smart watches, fitness bracelets, various toys, wireless speakers and headphones, flashlights, etc.), the requirement for stable and efficient power supplies is growing. Since the end of the last century, there has been an active development of energy-intensive lithium-ion batteries (LIBs), but now there are already problems of limited lithium resources and a predicted sharp rise in prices [1,2,3]. The price of sodium carbonate is 100 times less than the price of lithium carbonate; in addition, the world’s sodium reserves are much larger (~1000 times) than the ones of lithium. Despite the fact that the theoretical capacity of sodium is 1165 mA h g^−1^ (which is about three times less than for lithium), there is no reason to suppose that sodium-ion batteries (SIBs) will perform worse than LIBs. Several companies such as Aquion Energy (USA) and Faradion (UK) have already begun commercial production of SIBs [3,4]. Thus, increasingly more attention in the world is given to the development and improvement of SIBs with improved energy characteristics and an increased level of safety. In this regard, research is currently underway on more cost-effective materials for SIBs, one of the key components of which is electrolyte. The liquid or gel electrolytes [1,2,5] currently used in the mass production of batteries do not adequately guarantee the desired level of battery safety.

Polyelectrolytes with unipolar cationic conductivity are promising. In addition to unipolar conductivity, the advantages of such materials include flexibility, elasticity, and the possibility of thin-film fabrication and reliable contact at the electrode/electrolyte interface due to a good adhesion. Polyelectrolyte membranes swollen with nonvolatile high-boiling polar aprotic solvents can become a real alternative to the liquid and gel electrolytes.

**Figure 1:**
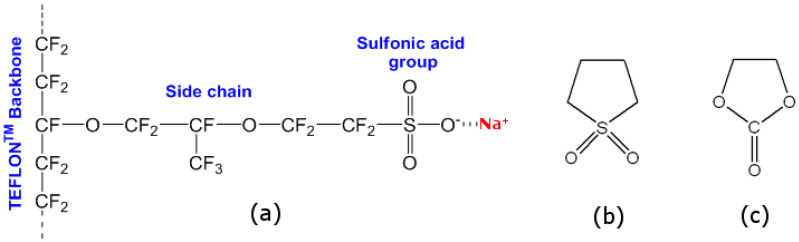
Structural formulas of (**a**) Na-Nafion, (**b**) sulfolane, and (**c**) ethylene carbonate.

Among a large variety of cation-conducting polyelectrolytes, of particular interest are Nafion-like membranes, which are a copolymer consisting of an electrically neutral base of polytetrafluoroethylene and a side ionogenic group of a perfluorinated sulfonic monomer associated with it (Figure 1a). The different nature of the bond in the main chain and the side group leads to a natural phase separation, which is enhanced by the introduction of a solvent. There are many models of the structure of Nafion, which are proposed on the basis of experimental and theoretical modeling data and are described in detail in the comprehensive review of Kusoglu and Weber [6]. These membranes have high mechanical, chemical, and thermal stability, wide operating temperature range and window of electrochemical stability, high value of proton conductivity, and the ease of conversion from the original acid form to various cationic ones [6,7]. In addition, these materials eliminate the need to use an additional structural material such as a separator, preventing direct contact and electronic transfer between the electrodes [7]. Along with its analogues, the perfluorinated sulfonic acid ionomer Nafion (developed by DuPont, USA) is commonly used as the ion-exchange membrane in fuel cells of various types [6,8,9,10,11], redox [12,13] and metal-ion [7,14,15,16,17,18] batteries, electrolyzers [19,20], sensors [21,22], etc.

A huge role in the characterization of the polyelectrolytes is played by the choice of the solvent for membrane plasticization. It affects indicators such as the temperature operating range, swelling degree, values of ionic conductivity, and fire and explosion safety.

To date, the best studied are polyelectrolytes for LIBs, which are used as a starting point in the study of post-lithium systems. Thus, the acceptable values of single-ion Li^+^ conductivity for practical usage should be at least 10^−5^ S cm^−1^ [23,24]. High ionic conductivity of the lithiated form of Nafion membrane (~10^−5^ and 10^−3^ S cm^−1^ [23,24,25,26]) was achieved by swelling the membranes with not only individual solvents (for example, amide solvents, carbonates, dimethyl sulfoxide, and dimethylformamide), but also their binary and ternary mixtures. The usage of mixtures helps to solve problems such as a narrow range of operating temperatures [25,27,28], low electrochemical stability [7,26,27], and dissolution of the polymer matrix at high temperatures [25]. Linear carbonates such as DMC, DEC, and EMC are frequently used as a cosolvent for ethylene carbonate; however, the problem of their flammability and volatility is still not solved [28]. Amide-containing solvents DMF, NMF, NMP, and DMA and their binary and ternary mixtures with carbonates have promising transport properties, but also a low electrochemical stability [26,27,28,29]. Highly polar DMSO, on the contrary, is stable in a wide range of potentials, but the membranes are unstable and have a phase transition in the region of 0 °C [27,28]. According to [30], the binary mixtures containing components with similar structure and melting temperature retain their liquid state at lower temperatures. The mixture of ethylene carbonate (EC) and sulfolane (SL) supports the statement: the solvent structures are obviously similar with the presence of electron donor groups capable of coordinating the sodium cation (Figure 1b,c); both solvents have high dipole moment (4.93 for EC and 4.81 for SL), low vapor pressure at *T* < 100 °C (0.003 and 0.009 kPa, respectively), and high flash points (145.5 ± 4 and 165 ± 4 °C) contributing to the safety of SIBs; the EC–SL system belongs to a simple eutectic type, with a eutectic point of −16 °C at 30 wt% EC, binary EC/SL mixtures are prone to transition into the metastable state of a supercooled liquid without crystallization of components (i.e., becoming glassy) [7,31]. In references [7,31,32], it was shown that binary mixtures of EC and SL are suitable and promising plasticizers for *lithiated* Nafion. For the best sample, single-ion lithium conductivity of 6 × 10^−6^ to 7 × 10^−4^ S cm^−1^ was achieved within a temperature range from −40 to +80 °C, with a wide electrochemical stability window of 0 to 6 V vs. Li/Li^+^. For this reason, these binary mixtures were chosen for the *sodium* form of Nafion.

Information on the conductivity of the sodium form of Nafion swollen with various aprotic plasticizers is too scarce, although its conductivity is not inferior to that of the lithium form, reaching values of 10^−3^ S cm^−1^ at room temperature [15,26,27,29,33,34]. There are also only a few works in which the electrochemical properties of cells with a polymer electrolyte based on the sodium form of Nafion were studied [15,33,34], in which the efficiency of the sodium-conducting Nafion membrane was shown.

Numerous experiments show that the ionic conductivity of the membrane increases with increasing the swelling degree [6]. This is probably due to the fact that most of the solvent molecules are in the free volume and are able to participate in the solvation of counterions, increasing their mobility. The swelling degree, in turn, is affected by the thermal history of Nafion, the properties of the plasticizer, type of counterion, and the saturation temperature [29,32,35]. Because the melting points of individual EC and SL are above room temperature (36.4 and 28.5 °C, respectively), it is advisable to saturate membranes with EC/SL binary mixtures at temperatures ≥ 40 °C when both components are in a liquid state.

Therefore, in this work, we studied the effect of mixing temperature (from 40 to 80 °C) on the swelling degree of the Na-Nafion membrane with the binary plasticizer EC–SL and, consequently, on other physicochemical and sodium-conducting properties.

## 2. Materials and Methods

### 2.1. Membrane Preparation

**Figure 2:**
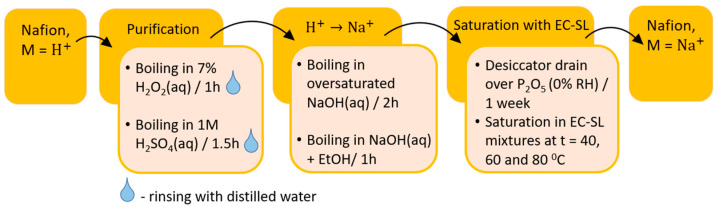
Scheme for obtaining the sodium form of Nafion membranes.

In this work, a commercial Nafion^®^ 115 (equivalent weight is 1100 g mole^−1^, thickness is 125 µm) (Du Pont de Nemours, Wilmington, DE, USA) extrusion membrane was used. The Na^+^ form of the membrane was prepared as described in our previous works [27,28] and shown in Figure 2 over the course of four consecutive stages: purification, neutralization by NaOH, thorough washing, and drying. The samples were first dried in air at 60 °C for 1 h and then in a desiccator at room temperature over P_2_O_5_ for seven days. Residual water content and the degree of proton substitution by sodium cations were monitored by infrared Fourier spectroscopy. 

All further manipulations of the Na-Nafion membrane and plasticizers were performed in an MBRAUN UNILAB glove box (MBraun Inertgas-Systeme, München, Germany) in an argon atmosphere (the content of H_2_O and O_2_ < 1 ppm).

### 2.2. Binary Plasticizer Preparation

A binary EC–SL plasticizer was prepared from anhydrous ethylene carbonate (99%) and sulfolane (99%), both from Sigma Aldrich (St. Louis, MO, USA). The molecular and physicochemical characteristics of individual solvents together with the phase diagram of EC–SL binary system are presented in [7,31]. The mass fraction of ethylene carbonate (*ω*(EC)) in the EC–SL mixtures varied from 0 to 1 with an increment of 0.1. Since the melting points of ethylene carbonate and sulfolane are above room temperature (≈ 36 and ≈ 29 °C, respectively), they were heated until wholly melted (up to 40 °C for EC and up to 30 °C for SL). Then, the mixtures were stirred for 30 min using a magnetic stirrer and kept at room temperature for at least 24 h before usage.

### 2.3. Membrane Swelling

The swelling of the Na-Nafion membrane was investigated gravimetrically. A dry membrane sample was placed in an excess volume of solvent (EC, SL, or EC–SL) over activated 3Å molecular sieves. Molecular sieves in this case were used to remove impurity amounts of water in the initial solvents and obtained electrolyte samples. To achieve an equilibrium degree of swelling, the samples were held under isothermal conditions (*T_sw_*) for 24 h (from 40 to 80 °C). The initial and final sample weights were measured. To remove the surface solvent, the samples were blotted dry before measurements. The swelling degree *W* (wt%) was calculated as:

*W =* (*m_sw_* − *m_dry_*)/*m_dry_* × 100%, (1)

where *m_dry_* and *m_sw_* are weights of the membrane samples after drying (see Section 2.1) and after swelling, respectively. The uncertainty of *W* measurement did not exceed 1–1.5%.

The molar uptake of solvent *λ* (equal to the number of solvents molecules per sodium cation) was calculated from *W* values using the formula:

*λ* = (*W* × EW)/(*Mr_solv_* × 100%), (2)

where EW is the equivalent weight of the dried Na-Nafion samples (equal to 1122 g mole^−1^) and *Mr_solv_* is the average molar mass of the used binary solvent. 

### 2.4. Thermal Stability

The thermal stability of the membranes was studied by simultaneous thermal analysis (STA) with mass spectrometric analysis of the gas phase on the device STA 409PC Luxx (NETZSH, Selb, Germany). The thermogravimetric analysis (TGA) and differential scanning calorimetry (DSC) curves were recorded under argon flow within a temperature range from 30 to 500 °C. The heating rate of the samples was 10 degrees per minute. 

Phase transitions were studied on the device Netzsch DSC 204 F1 Phoenix (NETZSH, Selb, Germany) within a temperature range from −70 to +70 °C. The heating rate of the samples was 10 degrees per minute. 

### 2.5. IR Spectroscopy

The method of IR spectroscopy was used to study intermolecular interactions and control the completeness of substitution of protons by sodium ions and the absence of water in the membrane samples. ATR IR spectra of the prepared samples were recorded under vacuum (<1 hPa) on spectrometer Vertex 70V (Bruker Corporation, Karlsruhe, Germany) at room temperature using a Bruker diamond attachment for the range 50–4500 cm^−1^ (resolution 4 cm^−1^, 16 scans). The spectra were registered with a single bounce Platinum ATR unit (Bruker Corporation, Karlsruhe, Germany) with Diamond crystal. At this scale, the spectra are normalized by the fixed depth of light penetration into the sample, thus ATR spectra in the distant IR region up to 50 cm^−1^ inclusive may be studied. Time of operation at which the hygroscopic sample was in the air along with the time required to transfer the sample into the attachment prior to start of evacuation of the cell holder of spectrometer was not more than 0.5 min.

### 2.6. AC Conductivity Measurements

Through-plane sodium conductivity was measured at a frequency range 0.1 Hz to 2 MHz using a P-5X analyzer (Elins LLC, Chernogolovka, Russia) with potential amplitude of 50 mV. The membrane samples (5 mm in diameter) were placed between two blocking Ti electrodes within a hermetically sealed two-probe test cell and equilibrated for 24 h at 25 °C before measurement. The electrolyte resistance *R_e_* was determined by intercepting the high-frequency part of the impedance curve with the real axis. The specific conductivity *σ* of the samples was obtained as follows:

*σ* = *d*/(*R_e_* × *S*) (3)

where *d* and *R_e_* are the thickness and resistance of the sample, respectively, and *S* is the area of electrical contact. The conductivity was determined by impedance spectroscopy with an error no more than 10%.

## 3. Results and Discussion

### 3.1. Solvent Uptake

**Figure 3:**
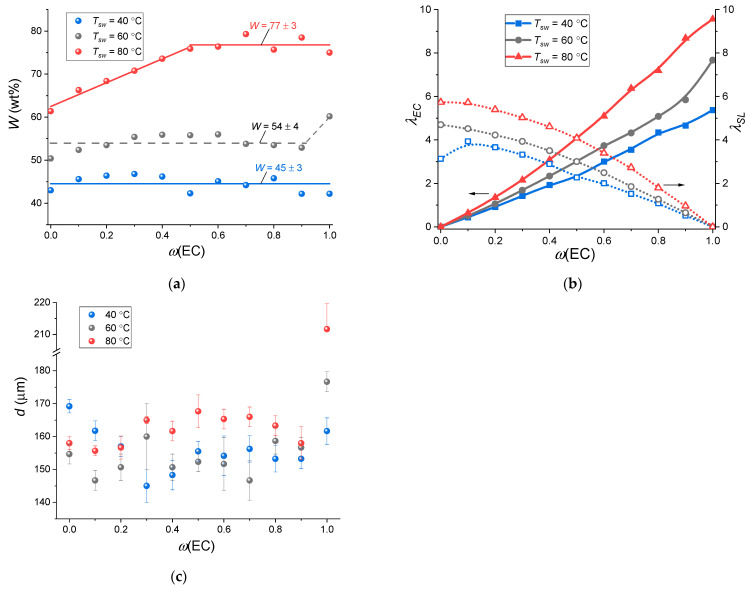
(**a**) Weight uptake, (**b**) molar solvent uptake, and (**c**) thickness of Na-Nafion membrane swollen at 40, 60, 80 °C as a function of *ω*(EC).

The membranes were saturated with EC–SL binary mixtures of varying composition at 40, 60, and 80 °C. The solvent uptake was defined by the gravimetric technique as described in Section 2.3. Figure 3a illustrates the dependence of the swelling degree *W* (calculated by Equation (1)) vs. *ω*(EC). It can be seen that for the lithiated form of Nafion [32], an increase in the temperature of swelling leads to an increase in *W*, although its value itself is much lower (for Li-Nafion *W* can exceed 200 wt% at *T_sw_* = 80 °C for pure EC), and the type of dependence on the composition of the plasticizing mixture is different for Na-Nafion. At *T_sw_* = 40 °C, the weight uptake does not depend on *ω*(EC) and equals to 45. At *T_sw_* = 80 °C, the swelling degree increases linearly with an increase in *ω*(EC) up to 0.5, and subsequently ceases to depend on the composition of the plasticizing mixture. At the swelling temperature of 60 °C, some intermediate state is observed: on the one hand, as for 40 °C, it does not seem to depend on the EC content (with the exception of *ω*(EC) = 1) and equals 54 wt%; on the other hand, at *ω*(EC) < 0.5, an increase in swelling degree is observed with an increase in EC content, as for *T_sw_* = 80 °C. Thus, the highest *W* relates to *ω*(EC) = 0.5–1 and reaches values of ~77 wt%. For Na-Nafion membranes saturated with binary and ternary mixtures of carbonates, dimethylacetamide, and tetrahydrofuran, *W* is even lower (20–30 wt% [29]) and does not exceed 65 wt% for the binary mixture of EC with propylene carbonate [34].

On the assumption that the mixed solvent composition absorbed by Na-Nafion is identical to the initial mixture used (no selective sorption), the total number of binary plasticizer molecules per Na^+^ cation in the swollen Na-Nafion membrane (molar uptake *λ*) was calculated using Equation (2). It is more convenient to use the number of molecules of each solvent per sodium ion, which were calculated as *λ_EC_* = *λ·ω*(EC) and *λ_SL_* = *λ·ω*(SL), respectively. Figure 3b shows that the *λ_EC_* value monotonically increases with an increase in *ω*(EC), while the λ values differ two-fold for pure solvents, *λ_SL_* = 3.1–5.7 and *λ_EC_* = 5.4–9.6.

In contrast to *W*, the membrane thickness does not change so clearly depending on the swelling temperature, although there is a tendency for growth with increasing *T_sw_* (Figure 3c). A significant effect is observed for pure EC: the membrane thickness increases from 162 to 212 μm with an increase in temperature from 40 to 80 °C. For membranes swollen with a mixture of EC–SL, the maximum thickness is observed for *ω*(EC) = 0.3–0.8 and is ~165 µm. The absence of a direct correlation between *W* and *d* apparently indicates a change not only in the thickness of the membranes, but also in their geometric dimensions in the other two coordinates. For example, the thickness of Na-Nafion membrane swollen in pure SL, decreases with increasing *T_sw_*, although the *W*-value increases. It can be associated with reorganization of transport channels and polymer matrix morphology due to the plasticization of the hydrophobic perfluorinated backbone of Nafion membrane. This phenomenon was observed during plasticization of the ammonium form of Nafion membrane with dimethyl sulfoxide [36].

### 3.2. Thermal Stability

**Figure 4:**
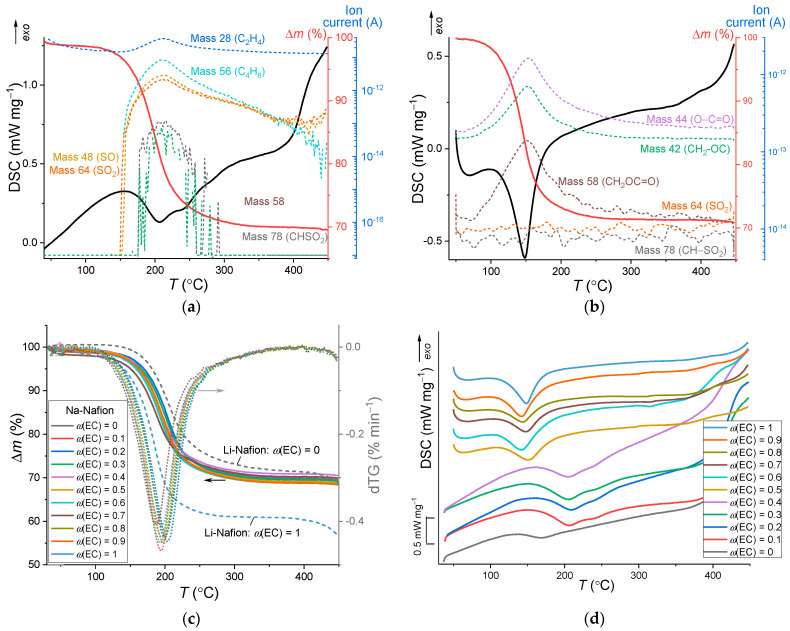

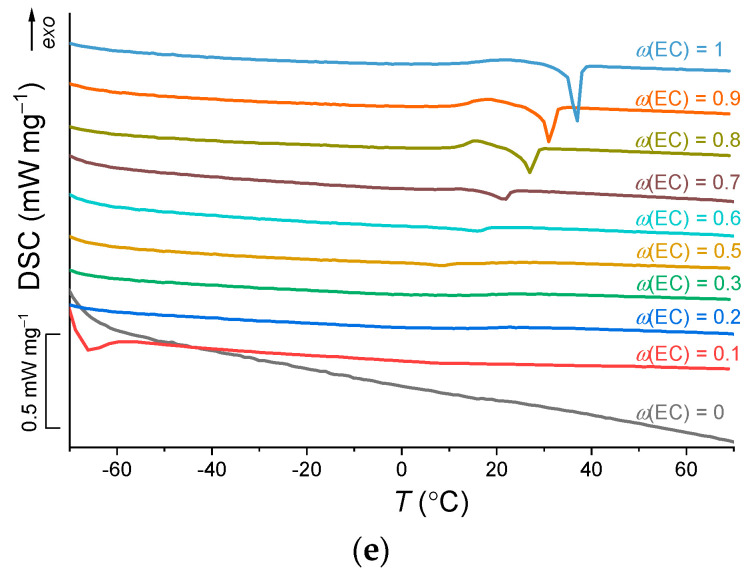
STA and ion current flow curves of the membrane swollen at 40 °C in (**a**) pure SL and (**b**) pure EC; (**c**) TGA curves (solid lines) and the first derivatives of the TGA curves (dotted lines) (for comparison, the TGA curves for the Li-Nafion are shown by dashed lines) and (**d**) DSC curves of the Na-Nafion membranes; (**e**) DSC curves in the low temperature region during heating.

Typical STA curves are shown in Figure 4a,b. For all the samples, one-step weight loss, associated with the process of the solvent removal from the Na-Nafion membrane in the flow of argon, is observed on the TGA curves up to ~300 °C and accompanied by an increase in the ion current of the residues of the initial solvents (ion current of masses 28–78 on Figure 4a–c). The absence of SO_2_ production in the region < 400 °C for the membranes swollen in pure EC (mass 64 in Figure 4b) confirms the absence of thermal degradation of the polymer matrix, which begins with the degradation of the side chain sulfonic acid groups. The other samples contain SL, the decomposition of which is also accompanied by the formation of SO and SO_2_ (mass 48 and 64), so it is impossible to separate the contributions to the ionic currents of the solvent and SO_3_ groups of the polymer. The single-stage process is confirmed by the presence of a single peak with maximum at ~200 °C on the first derivative of the TGA curves (dotted lines on Figure 4c). For the membrane swollen in the solvent mixture with *ω*(EC) = 0–0.5, the maxima of endothermic peaks lie in the range 140–170 °C and shift toward lower temperatures with increasing *T_sw_* (Figure 4d). For *ω*(EC) > 0.5, due to the higher boiling point of pure SL, the maxima of the endothermic peaks are shifted to the region of higher temperatures (~200 °C) and are not affected by the temperature of the membrane swelling. According to the DSC data for these samples, the evaporation process has two endothermic peaks. The content of plasticizer in the membranes obtained from TGA data coincides with that obtained from the membrane weighing within an error.

The absence of a current of ions with a mass of 48 and 64 for the sample with pure EC suggests that the entire weight loss is associated with the evaporation of the plasticizer from the membrane and not with its decomposition. The thermal decomposition of the polymer membrane was detected above 450 °C. The onset temperature of the thermal degradation of unswollen Na-Nafion is about ~150 °C higher than the one observed for the H-Nafion membrane [37]. Among the Nafion films exchanged with alkali metal, the sodium form has the highest values of the onset decomposition temperature (458 °C) in the series Li^+^–Cs^+^ [38]. It correlates well with the data obtained in our work: the lithium form of Nafion, plasticized with EC and SL, has a lower thermal stability compared to the sodium form (Figure 4c). It is worth noting that during the degradation of the unswollen cation forms of Nafion membrane SO_2_ production (mass 64) was not detected, which may be due to the formation of the ionic pair SO_3_^−^–Na^+^ that stabilizes the C–S bond [37,38]. In this work, ion currents of SO and SO_2_ (mass 48 and 64) are observed in Figure 4a,b in the region above 400 °C, which is probably due to the weakening of the C–S bond stabilization as a result of the coordination of solvent molecules to the Na^+^ cation.

The phase transitions of the solvent mixture in Na-Nafion membranes at low temperatures were studied by DSC for the membranes swollen at 40 °C. It was shown in reference [32] that a complete saturation of the lithiated form of Nafion with EC leads to a shift in the glass transition temperature of the membrane to below −80 °C. For Na-Nafion membranes swollen at 40 °C, no thermal transitions in the polymer are observed in the DSC curves (Figure 4e). For the samples with *ω*(EC) = 0.5–1, the curves show only the endothermic effect of the first-order phase transition associated with the melting of ethylene carbonate. As the EC content decreases, the melting enthalpy (Δ*H_m_*) decreases, and its maximum shifts toward low temperatures, from 36.4 to 9.1 °C (for Li-Nafion swollen in pure EC, this maximum is at the same temperature of 36.5 °C). The enthalpy of EC melting for the electrolyte swollen in pure EC is lower than for pure solvent (Δ*H*_0_ = 151.1 [39] or 153.3 J g^−1^ [40]). Low values of Δ*H_m_* can only be explained by the fact that some amount of EC does not freeze, for example, because of solvation, i.e., in the “bound” state. Similar phenomena are also known for water-saturated [41,42] and DMSO-saturated [27,36] Nafion membranes. Taking into account that no phase transitions are observed for a membrane saturated with pure SL in the studied temperature range, it can be assumed that only EC freezes. The mass fraction of freezing EC (*ω_F_*) can be determined from Equation (4) without considering the thermal effect of solvation and ion association processes:

(4) $$\omega_{F}=\frac{\Delta H_{exp}}{\Delta H_{calc}}\cdot 100\%,$$

where Δ*H_exp_* and Δ*H_calc_* are the experimental and theoretical enthalpy of fusion for the solvent at the temperature of phase transition (*T_ph_*).

Despite the fact that the values of Δ*H*_0_ and heat capacity of EC differ in the literature (for example, in [39,40]), it has little effect on the results of the calculation. That is why we calculated Δ*H_calc_* from the well-known Equation (5) [39]:

(5) $$\Delta H_{calc}=151.1-0.089\cdot \Delta T-0.079\cdot 10^{-3}\cdot \Delta T^{2},$$

where Δ*T* = 36.4 − *T_ph_*.

The results obtained indicate that even for pure EC, 90 wt% of the solvent is in the bound state and does not freeze (Table 1) in the temperature range studied. In terms of the number of molecules, only 0.5 EC molecules per sodium ion undergo a phase transition. Thus, five molecules of the solvent or EC–SL solvent mixture form the first coordination sphere. The loosely bound (bulk-like) solvent molecules constitute the second and higher coordination spheres of the ions.

### 3.3. Ionic Conductivity

**Figure 5:**
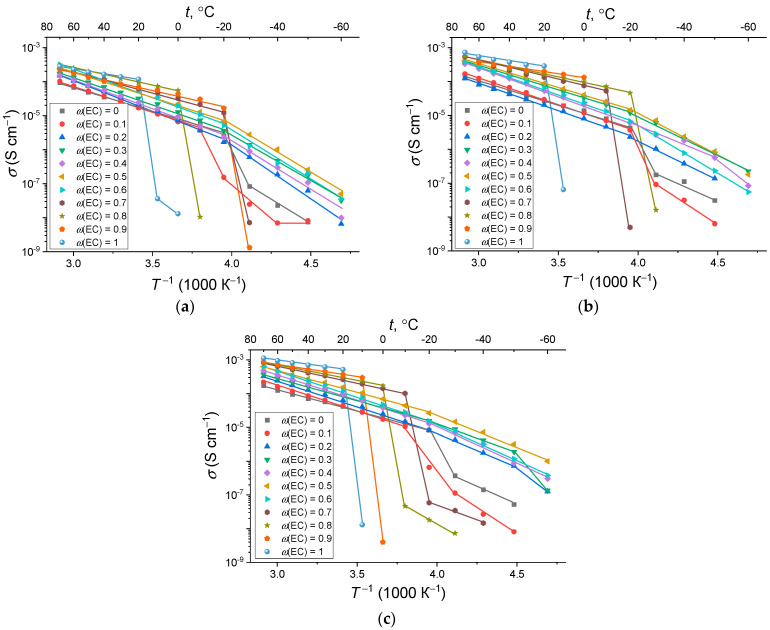
Temperature dependence of ionic conductivity of the Na-Nafion membranes, swollen at (**a**) 40 °C, (**b**) 60 °C, (**c**) 80 °C.

The results of conductivity measurements of swollen Na-Nafion membranes in the temperature range from −60 to +70 °C are shown on Figure 5. The ion conductivity has a linear dependence in the Arrhenius coordinates:

(6) $$\sigma =\sigma_{0}\cdot \exp\left(-E_{\sigma }/kT\right).$$

It can be seen that the membranes swollen in a mixture with *ω*(EC) = 0.2–0.6 retain their conductivity up to −60 °C, although they have a small kink in the region of −20 °C (probably associated with some structural rearrangement of the supercooled solvent in the membranes). The highest *σ*-value at −60 °C, reaching 10^−6^ S cm^−1^, is observed for the membrane with *ω*(EC) = 0.5 swollen at 80 °C. Thus, the sodium form of Nafion is not inferior to the lithiated one in conductivity [25], while liquid commercial electrolytes cannot operate at all at such low temperatures.

For the other membranes swollen with EC–SL mixture dominated by one or another solvent, the conductivity sharply deteriorates in the temperature range +20 to −20 °C due to reaching the freezing point of the solvent. For the membranes with high content of SL (*ω*(EC) = 0–0.1) the deterioration in conductivity is clearly not related to the freezing of the solvent (according to DSC data, there are no thermal transitions in this temperature range), but probably to a decrease in the mobility of ions. For Na-Nafion swollen in a binary mixture of carbonates, it was shown that at 60 °C the Na^+^ is almost exclusively coordinated by the solvent molecules, and at the lower temperature of 20 °C, the sodium ions are almost equally distributed between “solvent-coordinated” and “SO_3_^−^-bound” states, thus probably leading to a remarkable decrease in the sodium mobility [34]. Na-Nafion membranes swollen in pure EC have a minimal operating temperature range (above +20 °C) despite the highest conductivity at room temperature.

**Figure 6:**
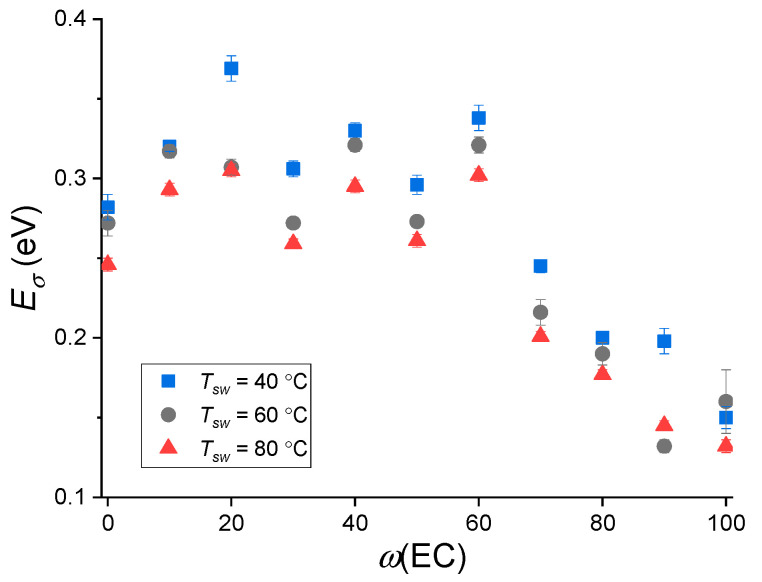
Dependence of the activation energy of conductivity on the EC content in mixture EC–SL above the kink temperature.

The calculation of the activation energy of conductivity *E_σ_* was carried out according to Equation (6) from the straight line slopes of temperature dependence of conductivity shown in Figure 5. Calculated values of *E_σ_* above the kink show a tendency to decrease by 10–20% with increasing *T_sw_* (Figure 6). This effect is expected, since at a higher swelling degree, the transport channels become wider and the mobility of the solvated Na^+^ ions increases. If we consider the influence of the plasticizing mixture composition, then two regions are observed for the *E_σ_* vs. *ω*(EC) dependence. In the first, with a high content of SL up to 0.4–0.5, the value of the activation energy does not depend on the composition of the solvents and equals to ~0.3 eV. With a further increase in the EC content, the value of *E_σ_* linearly decreases to 0.13–0.15 eV. 

**Figure 7:**
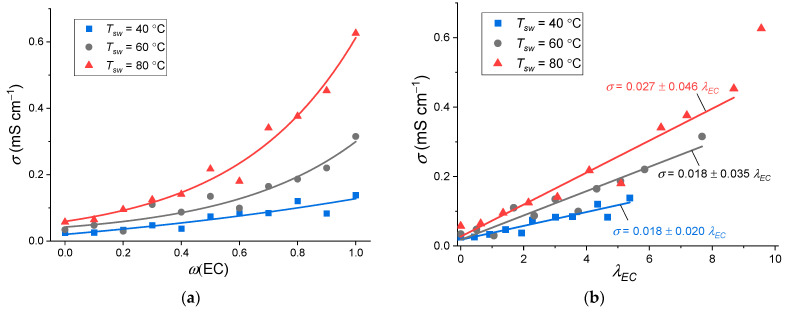

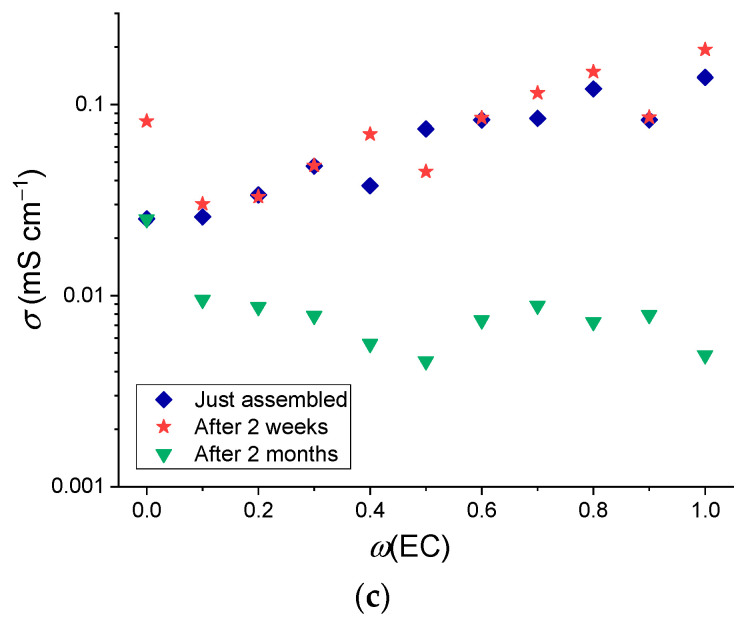
Ionic conductivity of the membranes at 30 °C for Na-Nafion membranes swollen at various temperatures as a function of (**a**) *ω*(EC) and (**b**) *λ_EC_*; (**c**) ionic conductivity of the membranes at 30 °C at various measurement times (*T_sw_* = 40 °C).

In the region of positive temperatures, the ionic conductivity of the studied membranes shows monotonous growth with an increase in the content of EC in the plasticizing EC–SL mixture (Figure 7a). At the same time, with an increase in *T_sw_*, the conductivity also increases, which, due to increasing *W*-value, may also indicate the widening of transport channels in Nafion membrane, which, in turn, improves the migration paths of Na^+^ cations. The ionic conductivity of Na-Nafion membranes swollen at 80 °C increases by an order of magnitude from 0.06 mS cm^−1^ (pure SL) up to 0.6 mS cm^−1^ (pure EC) at 30 °C. This monotonous behavior of the Na-Nafion membranes differ from the lithiated form of Nafion, whose maximum of ionic conductivity is observed at *ω*(EC) = 0.8 [7]. 

If we assume that the ionic conductivity is determined by the EC content, then the conductivity should depend linearly on *λ_EC_*. The ionic conductivity as a function of *λ_EC_* (Figure 7b), with the exception of the edge points, indeed has a linear character. However, at the same *λ_EC_* value, with an increase in *T_sw_*, an increase in the ionic conductivity is observed (the slope of the lines increases). Consequently, the swelling temperature affects the electrotransport properties of the resulting electrolytes not only due to an increase in the solvent content, but also for some other reasons.

The effect of storage time on membrane conductivity was studied for the example of Na-Nafion membranes swollen at 40 °C. The conductivity of the samples was measured at 30 °C immediately after assembly, after 2 weeks, and after 2 months. After 2 weeks, the conductivity for most of the samples did not deteriorate (Figure 7c). After 2 months, the conductivity decreased by about an order of magnitude for pure EC and, with the exception of pure SL, the conductivity value seemed to become independent from *ω*(EC). The drop in conductivity can be explained by the fact that during this time the samples were kept in measuring cells with a spring pressing. As a result, the membrane thickness decreased by 10–20% compared to the initial one, and, consequently, part of the solvent left the membrane, which led to deterioration in the migration of sodium ions.

### 3.4. Intermolecular Interactions

**Figure 8:**
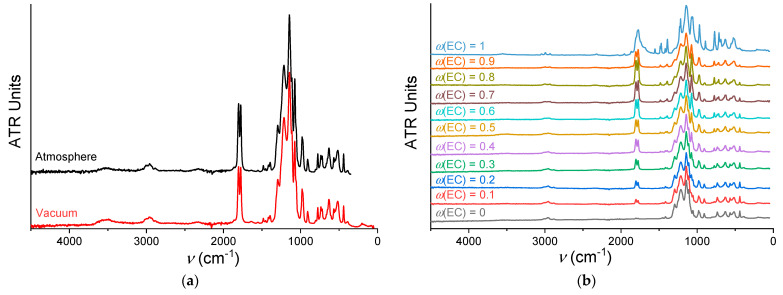
The survey FTIR ATR spectra of swollen Na-Nafion membranes: (**a**) recorded at various conditions (*ω*(EC) = 0.5, *T_sw_* = 40 °C); (**b**) with various *ω*(EC); swollen at 40 °C and swollen at 40 and 60 °C.

Initially, the spectra of a membrane sample swollen at 40 °C with *ω*(EC) = 0.5 were recorded at atmospheric pressure and under vacuum (Figure 8a). This was done to select the optimal experimental conditions under which the membrane would not absorb water from the air (–OH bond vibration band at ~3500 cm^−1^) and the solvent would not be removed from it. The spectra practically do not differ from one another, so it was decided to record the spectra under vacuum in order to exclude the accumulation of water from the air.

**Figure 9:**
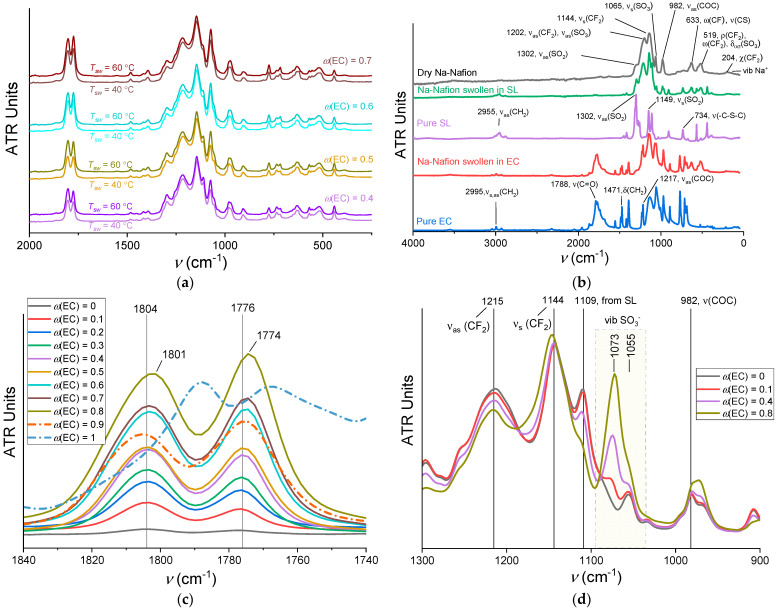
(**a**) The survey FTIR ATR spectra of Na-Nafion membranes swollen at 40 and 60 °C. (**b**) The survey FTIR ATR spectra of Nafion membrane dried over P_2_O_5_, initial solvents EC and SL and swollen Na-Nafion membranes. (**c**) Contours of the analytical band ν(C=O) of EC in the FTIR ATR spectra of swollen Na-Nafion. (**d**) Contours of the analytical band ν(SO) of the Na-Nafion (shaded area) in the FTIR ATR spectra of plasticized Na-Nafion.

The survey FTIR ATR spectra for a series of Na-Nafion membranes swollen at 40 °C with all compositions of EC–SL solvent mixtures are shown in Figure 8b. Increasing swelling temperature does not affect the spectra, at least over the temperature range in our experiment (Figure 9a). This shows that an increase in temperature does not cause a structural rearrangement of the membrane, and an increase in conductivity is associated exclusively with the widening of transport channels and an increase in the swelling degree. A change in the composition of solvent, on the contrary, noticeably affects the shape of the spectrum. For a detailed analysis, it is necessary to correlate the peaks and trace the dynamics of their change with an increase in the EC content in the solvent composition.

The assignment of vibration frequencies of the Na-Nafion membrane (Figure 9b) was carried out based on references [43,44,45]. The lowest frequency band (180–190 cm^−1^) refers to vibrations of sodium ions in the electrostatic field of sulfonic groups [46,47]. Note that the assignment of the bands in the range 900–1300 cm^−1^ is rather ambiguous, since the vibrational bands of the CF_2_ and SO_3_^−^ groups of the membrane and the solvent overlap. The change in the contours of the most significant bands depending on the composition of the EC–SL mixture in plasticized Na-Nafion membranes is shown in Figure 9c,d.

The intense band of ν(C=O) for uncoordinated EC solvent is observed in the carbonyl region at 1788 cm^−1^ while the peak at 1770 cm^−1^ stands for a Fermi resonance. The ν(C=O) frequencies in the plasticized membrane are slightly shifted to 1784 and 1773 cm^−1^, respectively (Figure 9b), which indicates the coordination of the EC molecule to the Na^+^ cation through the oxygen atom. With an increase in the EC content, the intensity of the band of stretching vibrations of the C=O bond is expected to increase (Figure 9c). The peaks slightly shift to the low-frequency region. This means that the vibration energy of the C=O bond decreases, which indicates an increase in the intermolecular bonds of this functional group with the surrounding molecules. At *ω*(EC) = 0.9, the peak of ν(C=O) becomes, on the contrary, less intense and slightly moves to low frequencies. These changes in FTIR spectra show good agreement with the theoretical calculation of stepwise Na^+^ solvation reactions in EC [48], where the peak of 5EC–Na^+^ slightly moves to low frequencies from 4EC–Na^+^ on the theoretical IR spectra due to the increase in average C=O bond length. It was shown that, in contrast to Li^+^, the larger Na^+^ and K^+^ ions show more disordered and flexible solvation structures [49]. In our systems, the number of EC molecules per sodium ions increases from 0.4 to 4.3 with increasing *ω*(EC) from 0.1 to 0.8, and equals to 4.7 and 5.4 at *ω*(EC) = 0.9 and 1 (Figure 3b). The large difference in the band contour for *ω*(EC) = 1 with the presence of three peaks (the third peak is observed in the region of 1802 cm^−1^) indicates the existence of both liquid EC in the pores of the membrane and solid EC on its surface.

To be clear, Figure 9d shows only four spectra in the region 900–1300 cm^−1^ for compositions with *ω*(EC) = 0, 0.1, 0.4, and 0.8. It can be seen that with an increase in the EC content, the intensities of the peaks at 1144 and 1215 cm^−1^, corresponding to symmetric and antisymmetric stretching vibrations of the CF_2_ group, change: the intensity of high-frequency peak decreases while the low-frequency one increases. The band of stretching vibrations of the C–O–C bond also becomes more intense and changes its contour, shifting to the low-frequency region with an increase in the EC content. From the perspective of these data, it can be concluded that the composition of the solvent affects the entire side chain, interacting with it electrostatically, and EC binds more strongly than SL. The corresponding SL peak at the frequency of 1109 cm^−1^ becomes less intense with a decrease in the SL content in the total amount of solvent. The contours of the ν(SO) bands located in the region 1030–1100 cm^−1^ change to a certain extent. The spectrum of the membrane kept in pure SL contains a single peak with the frequency 1055 cm^−1^ in this region. When EC is added, a second band appears with a maximum at 1073 cm^−1^, the intensity of which increases with an increase in *ω*(EC). It can be caused by a decrease in SO_3_^−^ polarization, which occurs due to more efficient solvation of Na^+^ ions by EC molecules and, accordingly, an increase in their mobility.

Additional complexity is imposed by the possibility of association of the solvent molecules. At 25 °C, the formation of EC-SL heteromolecular dimeric complex is more energetically probable and the formation of the SL-SL dimers is less favorable, and the probability of association of solvent molecules increases with decreasing temperature, while the probability of association of EC molecules also appears [7,31]. On the other hand, dimers form only in an excess of solvent, that is, when all sodium ions are solvated. Okoshi et al. [50] showed that the desolvation energy of Na^+^ in ethylene carbonate is lower than in sulfolane, i.e., all other factors being equal, Na^+^ ions are solvated precisely by EC molecules (confirmed by IR spectroscopy data in our work). In turn, SL molecules preferentially are in the state of dimers, interacting weakly with the side chains of the Na-Nafion membrane. Thus, for an optimal conductivity of Na^+^, a ratio of solvents is necessary so that the ions are solvated by EC molecules, while SL molecules are mainly in a free state. 

## 4. Conclusions

The influence of the swelling temperature of the Na-Nafion^®^ 115 membrane in a mixture of EC–SL solvents on the swelling degree, thermal stability, and cation-conducting properties of the resulting polymer electrolytes was studied. It was shown that an increase in the swelling temperature from 40 to 80 °C leads to an increase in the swelling degree of the membranes and an increase in conductivity within one order of magnitude due to the geometric expansion of pores and transport channels. Solvent evaporation begins in the range 140–250 °C, and degradation of the polymer matrix occurs above 450 °C. The first-order phase transition associated with the melting of ethylene carbonate is observed only in membranes in which the amount of solvent per sodium ion is greater than 5. According to IR spectroscopy data, EC molecules solvate Na^+^ ions more efficiently than SL molecules and reduce the polarization on the SO_3_^−^ group, contributing to an increase in the ion mobility. In this case, the addition of the fifth EC molecule changes the structure of the solvate shell of Na^+^. Thus, for the best conductivity, the ideal ratio of solvents would be some intermediate one, when Na^+^ ions are solvated by EC molecules, and SL molecules are mainly found in the free volume. It was established that membranes with *ω*(EC) = 0.2–0.6 have a wide operating temperature range from −60 to +70 °C. Among such electrolytes, the membrane with *ω*(EC) = 0.5 has the highest ionic conductivity: 10^−4^ S cm^−1^ at 30 °C and 10^−6^ S cm^−1^ at −60 °C. Therefore, it is possible to expand the operating temperature range of a sodium battery by varying the composition of the polymer electrolyte and the conditions for its preparation.

## Figures and Tables

**Table 1:** Calculated phase transition temperatures (*T_ph_*) and experimental values of the fusion enthalpy (Δ*H* *), fraction of freezing EC (*ω_F_*), number of freezing EC (*λ_EC_^F^*), and nonfreezing solvent (*λ^NONF^*) molecules in the sodium form of Nafion membrane swollen at 40 °C.

*ω*(EC)	*T_ph_*, °C	Δ*H_calc_*, J g^−1^	Δ*H_exp_*, J g^−1^	*ω_F_*, %	*λ_EC_^F^*	*λ^NONF^*
1	36.4	151.1	15.6	10.3	0.56	4.8
0.9	31.5	150.7	9.5	6.3	0.29	4.9
0.8	27.4	150.3	7.1	4.7	0.21	5.1
0.7	21.6	149.8	4.1	2.7	0.10	5.0
0.6	16.4	149.3	2.9	2.0	0.06	4.9
0.5	9.1	148.6	1.7	1.2	0.03	4.5

* Enthalpy values are given in J per gram of EC.

## Data Availability

The data presented in this study are available on request from the corresponding author.
